# Incomplete lung recovery following sub-acute inhalation of combustion-derived ultrafine particles in mice

**DOI:** 10.1186/s12989-016-0122-z

**Published:** 2016-02-24

**Authors:** A. Noël, R. Xiao, Z. Perveen, H. M. Zaman, R. L. Rouse, D. B. Paulsen, A. L. Penn

**Affiliations:** 1 Department of Comparative Biomedical Sciences, School of Veterinary Medicine, Louisiana State University, Skip Bertman Dr., Baton Rouge, LA 70803 USA; 2 Department of Pathobiological Sciences, School of Veterinary Medicine, Louisiana State University, Baton Rouge, LA 70803 USA; 3 Department of Anesthesiology, Columbia University Medical Center, New York, NY USA; 4 United States Food and Drug Administration, Silver Spring, MD USA

**Keywords:** Inhalation, Particulate matter, Combustion-derived ultrafine particles, Lung recovery, Inflammation, Oxidative stress, Biotransformation, Gene expression, Particle-laden macrophage biomarker

## Abstract

**Background:**

Particulate matter (PM) is one of the six criteria pollutant classes for which National Ambient Air Quality Standards have been set by the United States Environmental Protection Agency. Exposures to PM have been correlated with increased cardio-pulmonary morbidity and mortality. Butadiene soot (BDS), generated from the incomplete combustion of 1,3-butadiene (BD), is both a model PM mixture and a real-life example of a petrochemical product of incomplete combustion. There are numerous events, including wildfires, accidents at refineries and tank car explosions that result in sub-acute exposure to high levels of airborne particles, with the people exposed facing serious health problems. These real-life events highlight the need to investigate the health effects induced by short-term exposure to elevated levels of PM, as well as to assess whether, and if so, how well these adverse effects are resolved over time. In the present study, we investigated the extent of recovery of mouse lungs 10 days after inhalation exposures to environmentally-relevant levels of BDS aerosols had ended.

**Methods:**

Female BALB/c mice exposed to either HEPA-filtered air or to BDS (5 mg/m^3^ in HEPA filtered air, 4 h/day, 21 consecutive days) were sacrificed immediately, or 10 days after the final BDS exposure. Bronchoalveolar lavage fluid (BALF) was collected for cytology and cytokine analysis. Lung proteins and RNA were extracted for protein and gene expression analysis. Lung histopathology evaluation also was performed.

**Results:**

Sub-acute exposures of mice to hydrocarbon-rich ultrafine particles induced: (1) BALF neutrophil elevation; (2) lung mucosal inflammation, and (3) increased BALF IL-1β concentration; with all three outcomes returning to baseline levels 10 days post-exposure. In contrast, (4) lung connective tissue inflammation persisted 10 days post-exposure; (5) we detected time-dependent up-regulation of biotransformation and oxidative stress genes, with incomplete return to baseline levels; and (6) we observed persistent particle alveolar load following 10 days of recovery.

**Conclusion:**

These data show that 10 days after a 21-day exposure to 5 mg/m^3^ of BDS has ended, incomplete lung recovery promotes a pro-biotransformation, pro-oxidant, and pro-inflammatory milieu, which may be a starting point for potential long-term cardio-pulmonary effects.

## Background

Particulate matter (PM) is one of the six criteria pollutant classes for which National Ambient Air Quality Standards have been set by the United States Environmental Protection Agency [1]. PM is ubiquitous in outdoor air and contributes significantly to air pollution, especially in urban areas [2, 3]. PM are composed of ambient coarse (PM_10_, < 10 μm), fine (PM_2.5_, < 2.5 μm) and ultrafine (PM_0.1_, < 0.1 μm) particles. PM are found in multiple physicochemical forms, due to environmental as well as meteorological factors, and arise from diverse emission sources, both natural and anthropogenic [2, 4, 5]. Petroleum refineries, chemical plants and diesel engines release combustion-derived PM into the atmosphere following incomplete combustion of volatile hydrocarbons. The PM-rich soots produced during incomplete combustion of fuels are complex and may contain, in addition to polynuclear aromatic hydrocarbons (PAHs), a variety of oxygen radical-generating quinones and metal species [6–8]. PAHs, many of which are carcinogenic, are present in combustion-derived PM and thereby may increase considerably the potency of PM to induce adverse health effects [9]. Combustion-derived PM, particularly those resulting from traffic exhaust, are known health risk factors and are responsible for about 80 % of the human PM exposures [2]. Populations living in urban communities near heavily traveled highways potentially have a higher risk of exposure and consequently, of diseases related to air pollution [10]. In 2012, the World Health Organization estimated that outdoor air pollution accounted for 3.7 million premature deaths worldwide [11]. Thus, inhalation of outdoor air pollutants remains a leading public health concern [11].

Extended, high PM exposures result in sharp increases in morbidity and mortality. The Great London Smog of 1952 lasted 4 days, with peak particle concentrations reaching 0.4 mg/m^3^, and resulted in an estimated 12,000 deaths [2, 12]. In 1991, five months after the start of the fires at Kuwaiti oil depots and refineries, the concentration of ambient PM_2.5_ 20 km from the fire site still exceeded 830 μg/m^3^ [13]. In the 2001 World Trade Center (WTC) attack, people were exposed to complex particle mixtures from the destroyed towers and the burning aviation fuel. PM concentration levels remained above 1 mg/m^3^ more than one week after the attack [14]. Within a year of the WTC attack, a report on aggravated morbidity and mortality among ‘first responders’ was published [15]. PM-exposed firefighters also are a vulnerable sub-population of workers acutely exposed to very high concentrations of dust, fumes and gas mixtures. During the six days of the October 2003 Southern California wildfires, the maximum daily averaged PM_2.5_ concentration was 270 μg/m^3^ [16]. Furthermore, as in cases of pipeline sabotage, attacks on refineries (Gulf War I), accidents at refineries/oil storage depots (Texas City, TX, March 23^rd^ 2005; Buncefield, UK, December 13^th^ 2005) and routine flaring of volatiles at refineries, incomplete combustion of petrochemicals results in production of large particle-rich soot clouds. Although these sporadic events are considered to be local, large segments of specific populations are subject to these PM exposures. Thus, a variety of events result in sub-acute and sub-chronic exposure to high levels of airborne particles, with serious health problems detected in large numbers of people exposed. These real-life events are examples of acute and sub-acute exposures to high concentrations of PM in humans, and highlight the need to investigate the health effects induced by short-term exposure to elevated levels of PM, as well as to assess whether, and if so, how well these adverse effects are resolved over time.

To take into consideration the latest epidemiological and toxicological data, in 2012 the US EPA [17] revised the standards for particle pollution and established the annual mean particulate concentration at 50 and 12 μg/m^3^ for PM_10_ and PM_2.5_, respectively. According to the American Lung Association [18], nearly 15 % of all Americans are exposed acutely to unhealthy levels of airborne pollutants (>35.0 μg/m^3^). Each year, 200,000 excess deaths in the US are attributable to PM [19]. Epidemiological data have shown that air pollution is associated with decreased lung function in both healthy and susceptible populations of all ages [20–25]. It is now well established that the ultrafine component of air pollution causes adverse pulmonary effects through mechanisms associated with inflammation and oxidative stress [26–28]. Exposures to PM_2.5_ have been correlated with increased cardiopulmonary and lung cancer mortality [29, 30], as well as with increased risk of other respiratory and cardiovascular diseases [31–33]. Despite the risks to human health, there is insufficient information regarding the mechanisms underlying PM-mediated toxicity. Since many people are regularly exposed to PM, there is a need to investigate the link between epidemiology and biological plausibility by providing experimental data related to the potential mechanisms of pulmonary injuries caused by PM.

Earlier studies on pulmonary responses to ultrafine particle exposures in rodents [34–36] led us to investigate respiratory responses to mildly extended inhalation exposure to petrochemical combustion-derived ultrafine particles. Butadiene soot (BDS), generated from the incomplete combustion of 1,3-butadiene (BD), is both a model of PM mixture and a real-life example of a petrochemical product of incomplete combustion (i.e., soot) with the potential for environmental contamination and for adverse effects to human health [37, 38]. In New Orleans (LA) in 1987, BD leaked from a tank car and caught on fire. The tank car exploded and burned for 36 h. This resulted in the evacuation of thousands of people living in proximity to the area where the incident occurred. BD is carcinogenic and is listed among the forty most produced chemicals in the United States. BD is a high-volume, aliphatic hydrocarbon byproduct of petroleum refining and is used in the manufacture of synthetic rubber and other elastomers [39]. Similarly to diesel exhaust particles, BDS is an organic-rich mixture of inhalable fine and ultrafine (30–50 nm) carbonaceous particles, organized as chain-like aggregates of primary spherical particles, to which hundreds of PAH species, including benzo(a)pyrene [B(a)P] and other carcinogens, are adsorbed [2, 37, 38]. Inhalation of combustion-derived PM pollutants, especially those with PAHs adsorbed onto PM_2.5_ is a serious ongoing health concern [3]. In this study, we investigated the respiratory effects induced by BDS as an equivalent-alternative, relevant and under-investigated source and component of PM_2.5_.

We reported previously that limited inhalation exposures to BDS (5 mg/m^3^; 4 h/day, 4 days) leads to airway neutrophilia and epithelial damage, and to accumulation of particle-rich macrophages in airways of BDS-treated mice [40]. Microarray and confirmatory qRT-PCR analyses revealed sequential up-regulation of aryl-hydrocarbon receptor (*Ahr*) genes, oxidative stress response genes and pro-inflammatory genes [40]. Following *in vitro* exposure of a human bronchoepithelial cell line to BDS, we observed sequential up-regulation of the same gene sets identified in the *in vivo* exposures [41].

In this study, we hypothesized that moderately extended (21 days) inhalation exposures of mice to PM derived from BDS generation will instigate a suite of histopathologic and gene expression changes that will resolve gradually. From an inhalation toxicology perspective, this study addresses two questions:

1. What are the consequences to the lungs of moderately extended exposures (4 h/day, 21 consecutive days) to ultrafine particles derived from petrochemical combustion?
2. How quickly do the lungs recover?

## Results

### Soot aerosols characterization

**Fig. 1:**
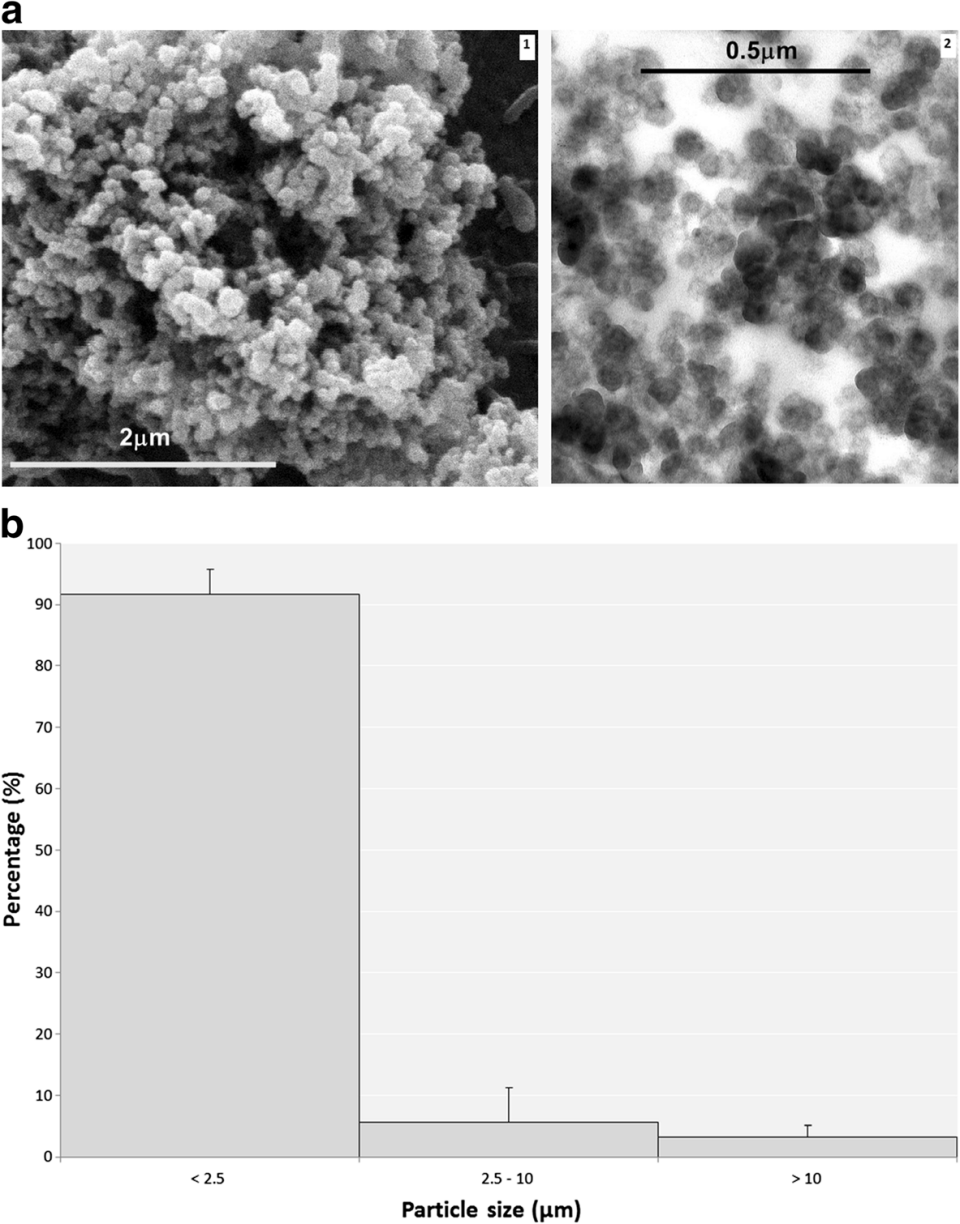
The combustion-derived BDS particle aerosols are composed mainly of PAH-rich (pyrene, acepyrene, anthracene and fluoranthene) fine sized (< PM_2.5_) agglomerated particles, (**a**) Electron microscopy images 1- scanning electron microscopy and 2- transmission electron microscopy) of BDS showing spherical, uniformly sized (20–40 nm diameter) primary particles, that have bundled into branches, chain-like shaped agglomerates that can reach the micrometer size range. **b** Particle size distributions of the aerosols show that > 90 % of the particles present are in the fine size range (PM_2.5_), and are composed of agglomerated individual ultrafine (<100 nm) particles [38]

PAHs comprise 16–20 % of BDS particles by weight, with pyrene, acepyrene, anthracene, and fluoranthene, being the four predominant PAHs. Detailed physical and chemical analyses of BDS were presented previously [37, 38]. A scanning electron microscope image of the aerosolized BDS particles is shown in Fig. 1a1. The ultrafine BDS particles behave similarly to other nanoparticles and aggregate into longer particles (Fig. 1a2). Particle size distribution of the soot aerosols showed that 90 ± 4.1 % (Mean ± SD) of the particles present were in the fine size range (PM_2.5_; Fig. 1b). Thus, the mice were exposed to BDS aerosols composed of PAH-rich nanoparticles.

### Inflammatory responses and BDS retention in exposed lungs are apparent

#### BALF cytology, BDS retention and lung histopathology

**Fig. 2:**
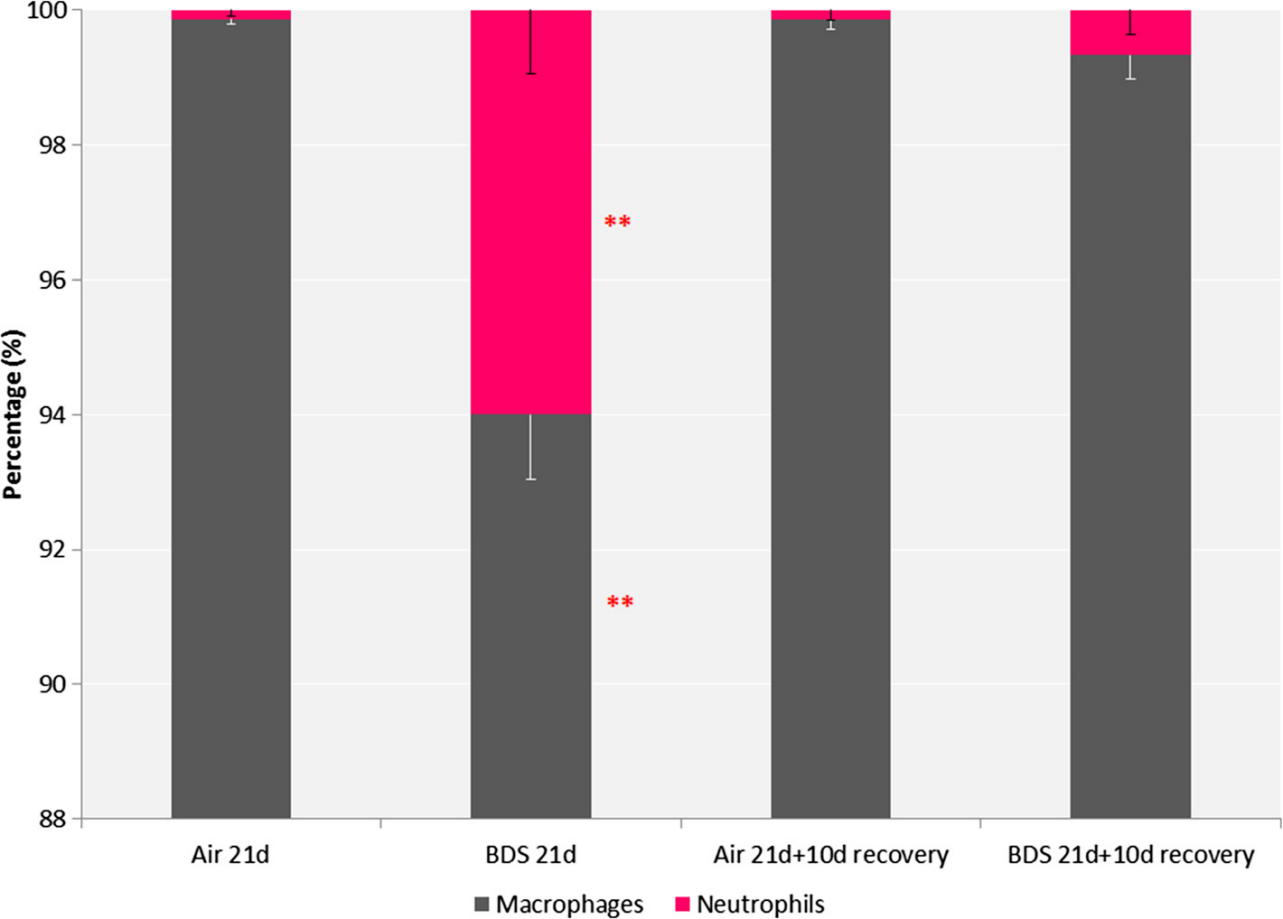
21 days exposure to BDS significantly increased the percentage of neutrophils (6 % ± 0.95) in BALF; following the 10-day recovery period, the percentage of neutrophils decreased to just above pre-exposure levels (0.67 % ± 0.37). No eosinophils were observed. Data represent the mean ± the standard error of the mean (SEM) for *n* = 6–8 mice per group. ANOVA followed by a Tukey’s test, *p* < 0.05: ** BDS 21d statistically different from all other groups

Lung responses of mice exposed to BDS particles (5 mg/m^3^, 4 h/day, 21 consecutive days) mixed with HEPA-filtered air were compared to lung responses of HEPA-filtered air controls. Groups of mice were sacrificed immediately after the 21-day exposures ended (BDS 21d) and after 10 days of recovery (BDS 21d + 10d recovery). Differential 300-cell leukocyte counts were performed on lung lavage samples from 6–8 mice/group. As expected, macrophages predominated in all the lavage samples (Fig. 2); however, in the BDS 21d mice, the BALF differentials showed a mild but significant (*p* < 0.05) elevation of the percentage of neutrophils (6 % ± 0.95), compared with controls, which were virtually neutrophil-free (0.13 % ± 0.08). Eosinophils were not detected in any of the differential counts. Neutrophil differential values were significantly reduced (0.67 % ± 0.37) in the BDS 21d + 10d recovery group compared to the BDS 21d group, showing that the increase in the percentage of neutrophils was consistent with a transient inflammatory reaction in the lungs (see below).

**Fig. 3:**
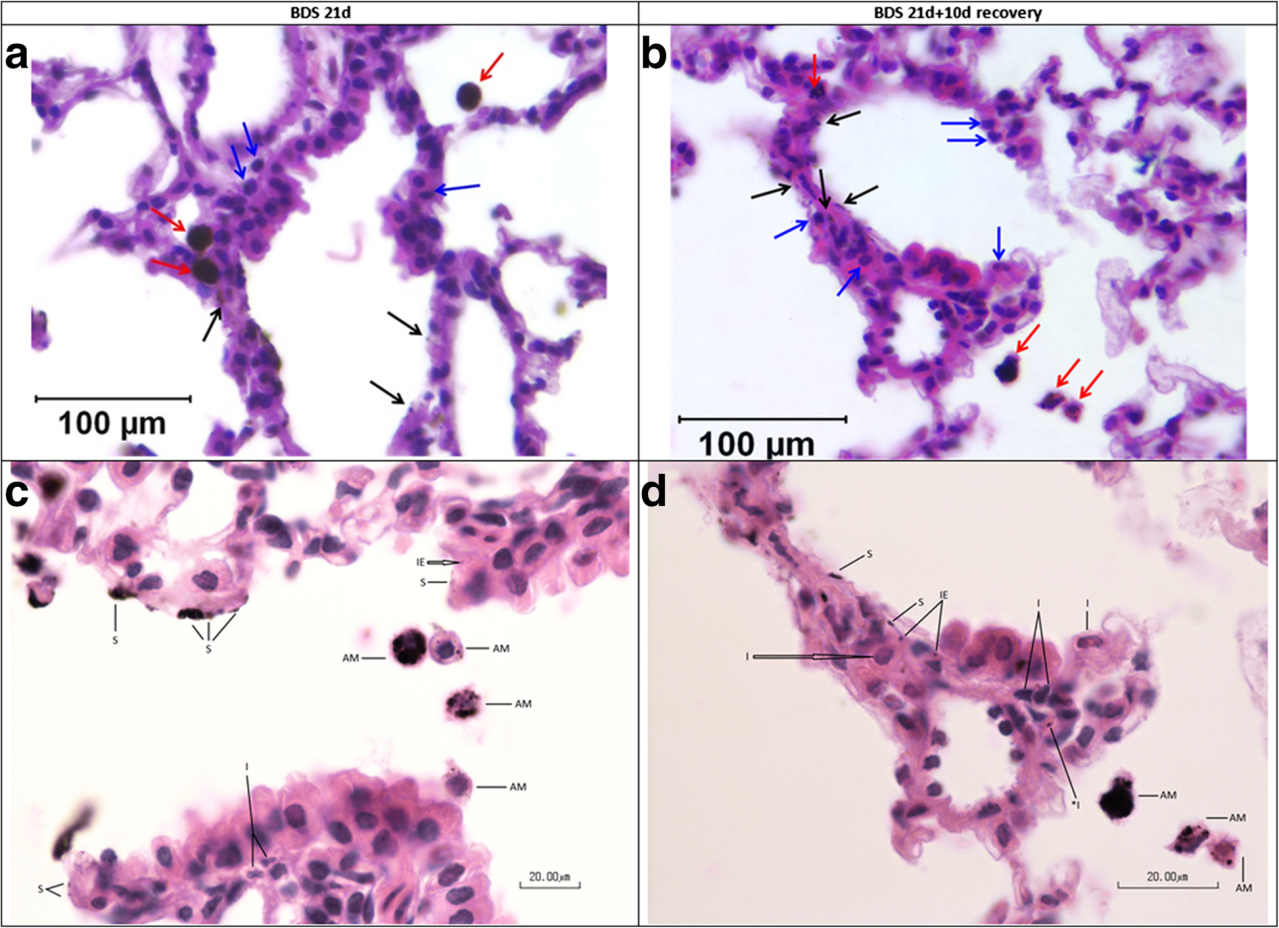
All mice from exposed groups had intracellular BDS. Mice were sacrificed immediately after the last exposure ended (**a** and **c**) or after 10 days of recovery (**b** and **d**). Red arrows = BDS-filled macrophages; blue arrows = inflammatory cells; black arrows = intracellular BDS; AM = alveolar macrophage containing phagocytized BDS; I = interstitial inflammatory cells; *I = BDS in interstitial inflammatory cell; IE = intraepithelial BDS; S = BDS apposed or adhered to mucosal surface

Lungs appeared grossly similar at both time points (BDS 21d vs BDS 21d + 10d recovery). Soot-filled macrophages (red arrows) were prominent in the airways of both groups of BDS-exposed mice, and the numbers of soot-filled macrophages were not appreciably different between the two BDS-exposed groups (Fig. 3a, b). Soot particles (black arrows, Fig. 3a, b), not taken up by macrophages, were present in the airways of both groups, as well as immediately adjacent to airway epithelial cells. In both groups of exposed mice, BDS particles were taken up by airway cells. Inflammatory cells along the airways (blue arrows, Fig. 3a, b) were present in both BDS-exposed groups. Neither soot particles (macrophage-bound or free) nor inflammatory cells were detected in lungs of control mice (data not presented). Patterns of soot particle deposition and retention in mouse lungs were similar, both immediately after a 21-day continuous exposure ended and after 10 days of recovery.

**Fig. 4:**
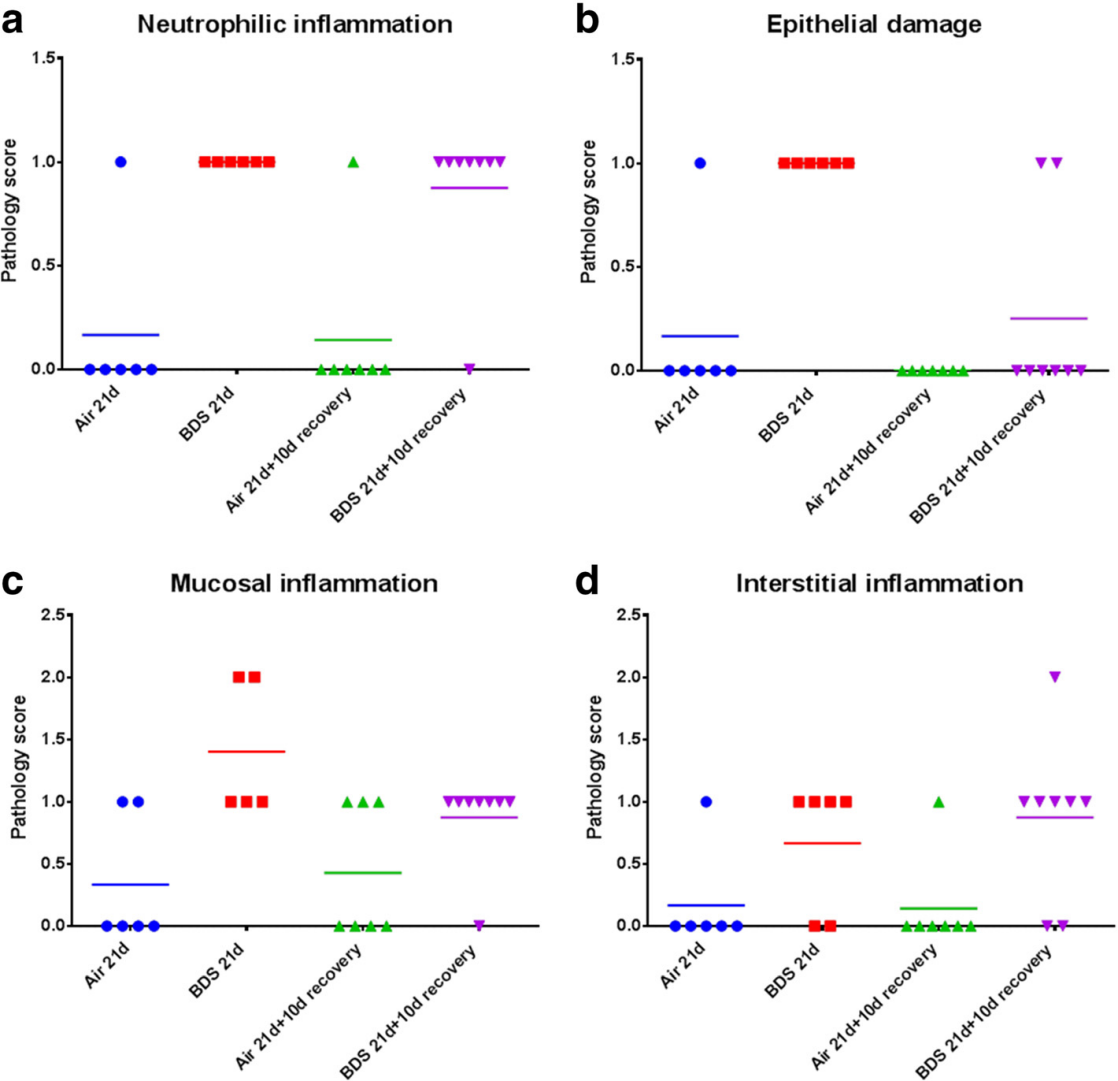
Pathology evaluation of the lungs confirms that the mild neutrophilic inflammation is caused by 21 days of exposure to BDS. **a**, **c**, **d** specific type of inflammation present in the lungs; (**b**) epithelial damage observed in the lungs. 5–8 mice per group. Scoring: 0 = none; 1 = mild; 2 = moderate; 3 = severe

Histopathology evaluation of lung sections revealed the presence of mild airway inflammatory responses immediately following 21 days of BDS exposure that persisted after 10 days of recovery (Fig. 4a, c, d). The outcomes measured included mucosal, interstitial and alveolar inflammation, presence of airway neutrophilia and epithelial cell damage. Neutrophils were present in mice in both BDS exposure groups (Fig. 4a). Despite the similarities in lung particle burden (Fig. 3a & b) and lung neutrophilia (Fig. 4a) seen in BDS 21d and BDS 21d + 10d recovery mice, neutrophils were essentially absent from BALF after 10 days recovery (Fig. 2). Among all the tissue outcomes, only epithelial damage was reduced in the 10-day recovery samples (Fig. 4b). Thus, soot particles were not cleared from the lungs (Fig. 3b), and inflammatory cells were still present in the lungs (Fig. 4a), 10 days after inhalation of soot particles had ended. Control airways had no detectable particles and showed no evidence of inflammation (Fig. 4a, c, d).

**Fig. 5:**
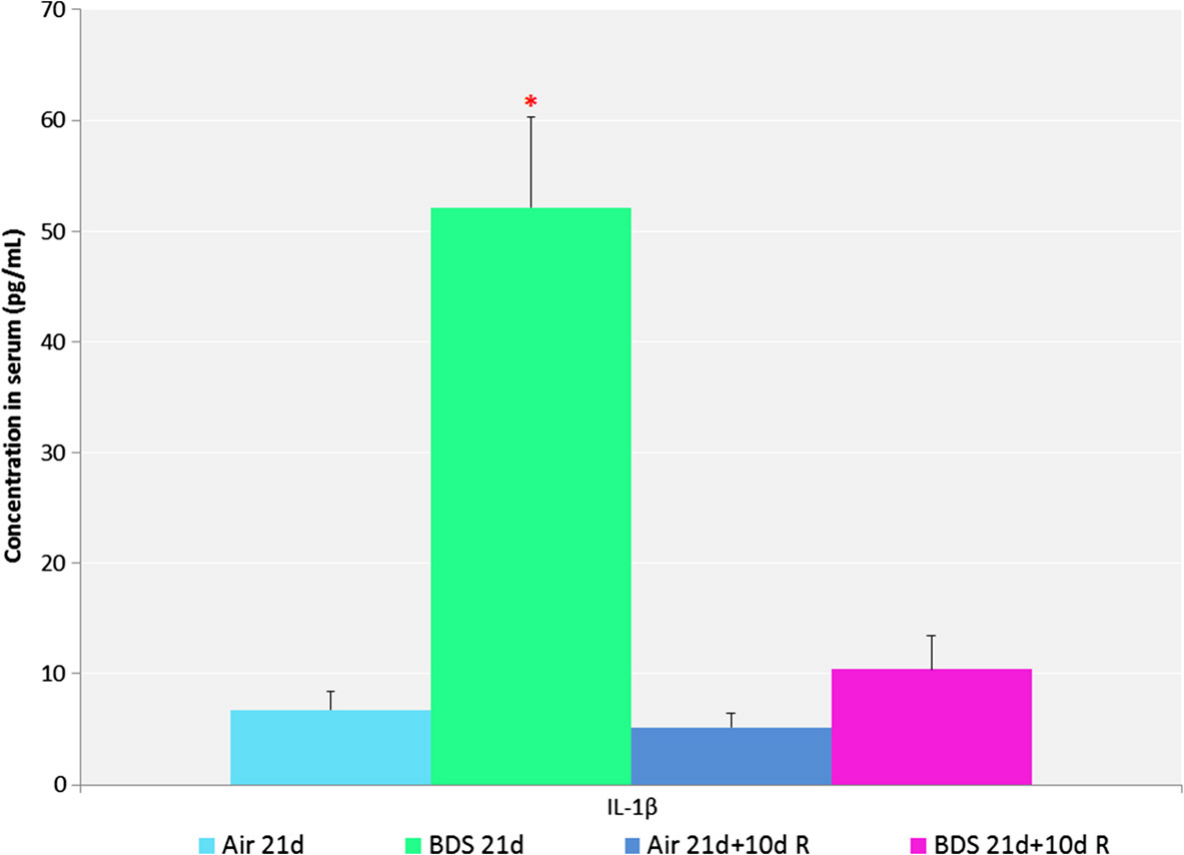
21 days of exposure to BDS significantly increased the BALF concentrations of IL-1β. Data represent the mean ± the standard error of the mean (SEM) for *n* = six mice per group. ANOVA followed by the Holm-Sidak method for all pairwise multiple comparisons, *p* < 0.05: *BDS 21d statistically different from all other groups

Inflammation markers in BALF were analyzed to assess levels of airway inflammation following a 3-week exposure to BDS ultrafine particles. Out of the 7 pro-inflammatory cytokines analyzed, only KC and IL-1β had levels above the limit of detection. The 3-week exposure to BDS induced a significant increase in IL-1β compared to air exposures or BDS 21d + 10d recovery (Fig. 5). Therefore, a pro-inflammation airway cytokine response marked by IL-1β was detected in BALF (Fig. 5) concurrent with the inflammatory reaction observed in the lungs of BDS-exposed mice but not BDS-exposed mice allowed 10 days of recovery (Figs. 2 and 4a, c, d).

### BDS-associated changes in gene expression are detected even 10 days after exposures end

#### Microarray

Principal component analysis of the microarray data revealed distinct clustering by treatment group, with greater variation between groups than within groups. This validated the subsequent intergroup comparisons.

**Fig. 6:**
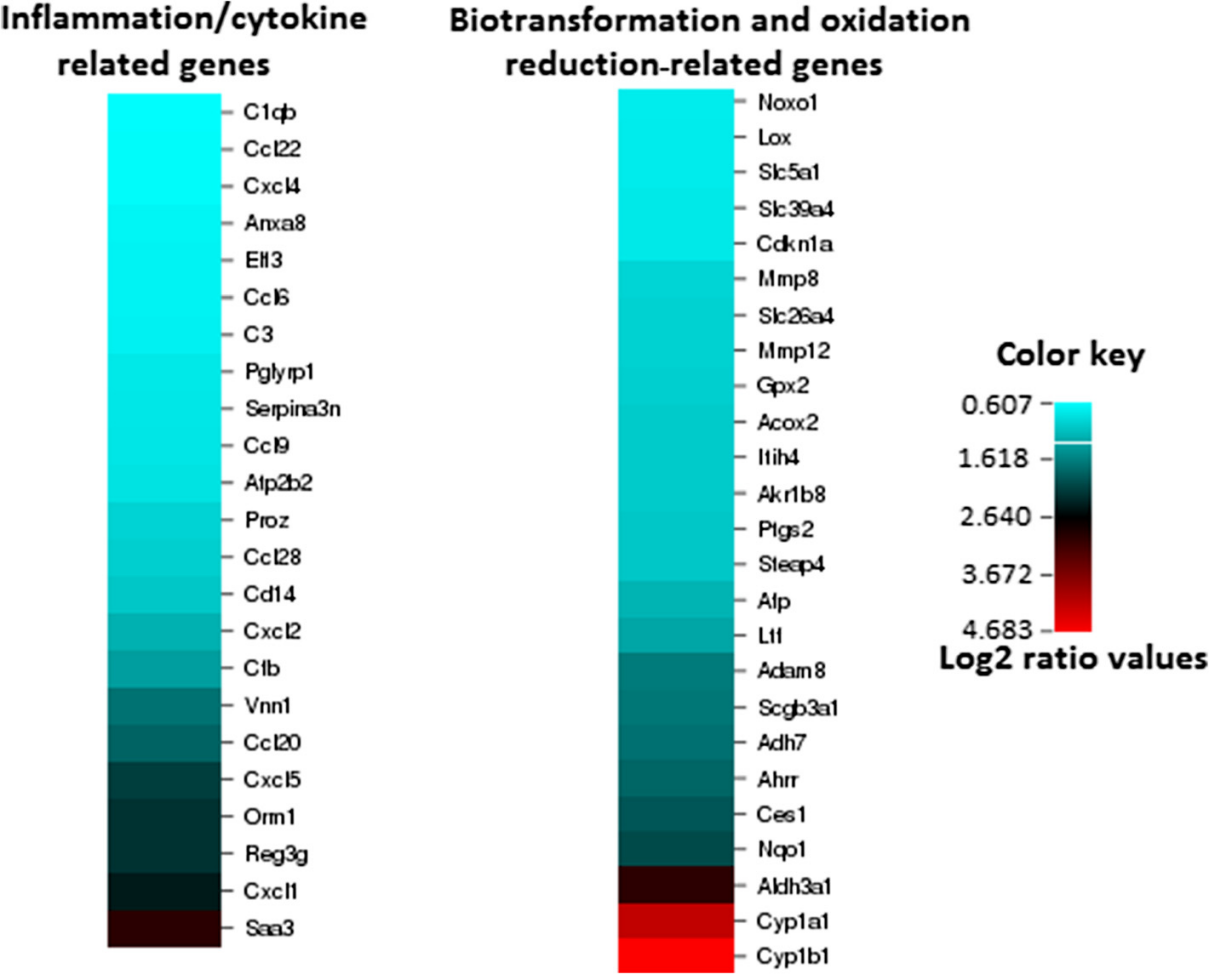
21 days exposure to BDS significantly increased the expression of several inflammation, cytokine, biotransformation and oxidation reduction-related genes relative to controls. Microarray results are presented for global gene expression of left lung (see methods for details regarding the gene expression analysis). Data are expressed as BDS 21d compared to controls (log2 ratio fold increases) for selected genes (*n* = four mice per group)

Next, microarray results were examined to determine whether genes were differentially expressed in either BDS group vs. controls. After Expression Analysis filtering of the microarray data (fold change ≥ 1.5, FDR and transcript *p*-value each < 0.05) and removing duplicate, poorly characterized and unmapped transcripts, 102 unique transcripts (all up-regulated) remained that were differentially-expressed in lungs of BDS 21d mice vs. lungs of control mice. Three distinct groups of genes - biotransformation, oxidative stress, inflammation (Table 1) - were apparent from among the 102 unique up-regulated transcripts (BDS 21d vs. control) that passed all the selection criteria. Up-regulated Phase I biotransformation genes included *Cyp1a1* (18.32 fold) and *Cyp1b1* (25.83 fold); aryl hydrocarbon receptor repressor, *Ahrr* (3.56 fold) and *Aldh3a1* (7.97 fold). Up-regulated oxidative stress response genes included, *Nqo1* (4.16 fold); glutathione peroxidase 2, *Gpx2* (1.97 fold), and *carboxylesterase 1* (3.88 fold). Inflammatory/immune response genes included chemokine (C-X-C motif) ligand 2, *Cxcl2* (2.33 fold), *Cxcl5*, the mouse homolog of the human chemokine *Cxcl6* (4.43 fold); *Ccl20* (3.60 fold); *Ccl28* (1.96 fold); a disintegrin and metallopeptidase, domain 8, *Adam8* (3.13 fold); *Vanin 1* (3.30 fold); prostaglandin-endoperoxide synthase 2, *Ptgs2* (2.05) and the acute phase proteins, ceruloplasmin, *Cp* (1.82); complement factor B, *Cfb* (2.61); orosomucoid 1, *Orm1* (4.73 fold); and serum amyloid a3, *Saa3* (7.86-fold). Thus, microarray analysis reveals that 3 weeks of daily inhalation exposure to BDS particles results in up-regulation of biotransformation, oxidative response, and inflammatory genes (Fig. 6).

**Table 1:** Function-related clusters of differentially expressed genes for 21d BDS mice sacrificed immediately after the last exposure ended vs. air controls (fold change > 2 and FDR *p*-value < 0.05)

Gene symbol	Gene descriptor	Raw estimated fold change BDS 21d vs air control
	Inflammation cluster	
*Saa3*	serum amyloid A 3	7.86
*Cxcl1*	chemokine (C-X-C motif) ligand 1	5.42
*Reg3g*	regenerating islet-derived 3 gamma	4.75
*Orm1*	orosomucoid 1	4.73
*Cxcl5*	chemokine (C-X-C motif) ligand 5	4.43
*Ccl20*	chemokine (C-C motif) ligand 20	3.60
*Vnn1*	vanin 1	3.30
*Adam8*	a disintegrin and metallopeptidase domain 8	3.13
*Cfb*	complement factor B	2.61
*Cxcl2*	chemokine (C-X-C motif) ligand 2	2.33
*Cd14*	CD14 antigen	2.07
*Ptgs2*	prostaglandin-endoperoxide synthase 2	2.06
*Ccl28*	chemokine (C-C motif) ligand 28	1.96
	Biotransformation cluster	
*Cyp1b1*	cytochrome P450, family 1, subfamily b, polypeptide 1	25.83
*Cyp1a1*	cytochrome P450, family 1, subfamily a, polypeptide 1	18.32
*Aldh3a1*	aldehyde dehydrogenase family 3, subfamily A1	7.97
*Ahrr*	aryl-hydrocarbon receptor repressor	3.56
*Adh7*	alcohol dehydrogenase 7 (class IV), mu or sigma polypeptide	3.35
*Scgb3a1*	secretoglobin, family 3A, member 1	3.21
*Ltf*	lactotransferrin	2.50
*Afp*	alpha fetoprotein	2.31
*Steap4*	STEAP family member 4	2.07
*Itih4*	inter alpha-trypsin inhibitor, heavy chain 4	2.02
	Oxidative stress cluster	
*Nqo1*	NAD(P)H dehydrogenase, quinone 1	4.16
*Ces1*	carboxylesterase 1	3.88
*Akr1b8*	aldo-keto reductase family 1, member B8	2.02
*Acox2*	acyl-Coenzyme A oxidase 2, branched chain	2.01
*Gpx2*	Glutathione peroxidase 2	1.97

Ten days after BDS exposures ended, many of the genes that were up-regulated in lungs of BDS 21d mice (above) remained significantly elevated although the absolute levels of expression were reduced (Table 2). Phase I and phase II biotransformation genes in this group included *Ahrr* (2.14 fold), *Cyp1b1* (2.60 fold), and *Aldh3a1* (1.72 fold); the inflammatory/immune response genes *Il-6* (2.08 fold), *Adam8* (1.82 fold) and *Ptgs2* (1.85 fold); and the acute phase proteins *Saa1* (1.69 fold), *Orm2* (2.17 fold) and *Cfd* (3.53 fold). Thus, even 10 days after BDS exposures end, biotransformation, oxidative stress and inflammation-related genes remain significantly high (vs. controls) in the lungs of BDS-exposed mice (Table 2).

**Table 2:** Selected differentially expressed genes for BDS 21d + 10d recovery vs. air controls (fold change > 1.5 and 2 and FDR *p*-value < 0.05)

Gene symbol	Gene descriptor	Raw estimated fold change BDS 21d + 10d R vs air control
*Saa1*	serum amyloid A 1	1.69
*Aldh3a1*	aldehyde dehydrogenase family 3, subfamily A1	1.72
*Adam8*	a desintegrin and metallopeptidase domain 8	1.82
*Ptgs2*	prostaglandine-endoperoxide synthase 2	1.85
*Clec4g*	C-type lectin domain family 4, member G	2.01
*Dnai2*	dynein, axonemal, intermediate chain 2	2.03
*Retn*	resistin	2.06
*Il-6*	interleukin 6	2.08
*Ahrr*	aryl-hydrocarbon receptor repressor	2.14
*Tmem45b*	transmembrane protein 45B	2.15
*Dmrt2*	doublesex and mab-3 related transcription factor 2	2.17
*Orm2*	orosomucoid 2	2.17
*Cidea*	cell death-inducing DFFA-like effector a	2.20
*Cxcr6*	chemokine (C-X-C motif) receptor 6	2.22
*Prmt8*	protein arginine methyltransferase 8	2.29
*Cyr61*	cysteine-rich, angiogenic inducer, 61	2.40
*Slc6a20*	solute carrier family 6 (proline IMINO transporter), member 20	2.41
*Sult1d1*	sulfotransferase family 1D, member 1	2.48
*Fabp4*	fatty acid binding protein 4, adipocyte	2.51
*Ighm*	immunoglobulin heavy constant mu	2.56
*Cyp1b1*	cytochrome P450, family 1, subfamily B, polypeptide 1	2.60
*Fabp4*	fatty acid binding protein 4, adipocyte	2.89
*Sult1d1*	sulfotransferase family 1D, member 1	3.07
*Thrsp*	thyroid hormone responsive	3.13
*Cfd*	complement factor D (adipsin)	3.53
*Adipoq*	adiponectin, C1Q and collagen domain containing	3.77
*Plunc*	palate, lung and nasal epithelium associated	4.58
*Ca3*	carbonic anhydrase III, muscle specific	4.84

#### qRT-PCR

**Fig. 7:**
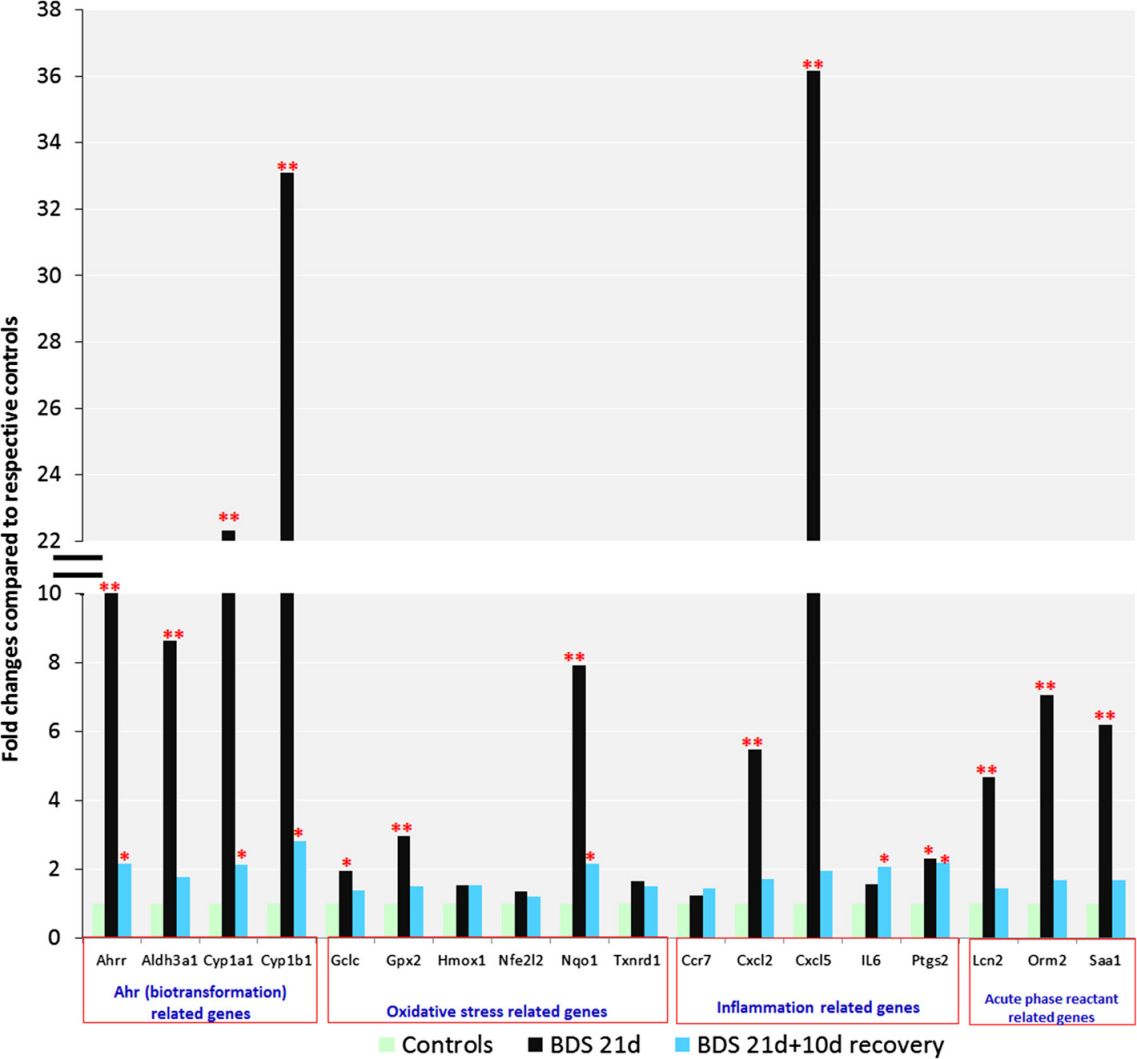
qRT-PCR results confirm that 21 days exposure to BDS significantly increases the expression of several biotransformation, oxidative stress, inflammation and acute phase reactant- related genes. The 10 day recovery period allows a significant decrease in those gene expression levels. Data represent the mean for *n* = 5–7 mice per group. UNIVARIATE and TTEST procedures, *p* < 0.05: ** BDS 21d statistically different from respective controls and BDS 21d + 10d recovery. *BDS 21d or BDS 21d + 10d recovery statistically different from respective controls

Figure 7 shows results obtained by qRT-PCR that were used to a) confirm up-regulation of 4 biotransformation (*Ahrr*, *Aldh3a1*, *Cyp1a1* and *Cyp1b1*), 6 oxidative stress (*Gclc*, *Gpx2*, *Hmox1*, *Nfe2l2*, *Nqo1*, *Txnrd1*) and 8 inflammation genes (*Ccr7*, *Cxcl2*, *Cxcl5*, *Il6*, *Ptgs2*, *Lcn2, Orm2, Saa1*) in BDS 21d vs control mice and b) determine whether those genes remained up-regulated (vs. controls) in BDS 21d + 10d recovery mice. These data confirmed the array results.

#### Western blotting

**Fig. 8:**
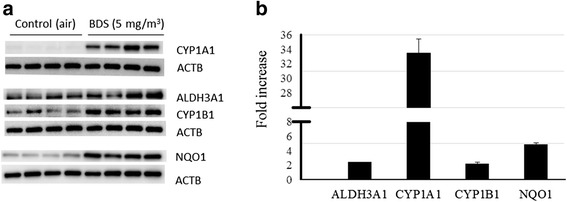
Protein expression: (**a**) Western blots confirmed that ALDH3A1, CYP1A1, CYP1B1, and NQO1 were up-regulated in Balb/c mice exposed to 5 mg/m^3^ of BDS 4 h/day for 4 days. 5 mg/m^3^ BDS treatment vs. controls. **b** Mean densitometry ± SEM results showing fold increases of treated mice vs. controls for the four proteins analyzed

Figure 8 demonstrates that protein expression confirmed qRT-PCR and gene expression results, showing that ALDH3A1, CYP1A1, CYP1B1, and NQO1 were up-regulated in BALB/c mice exposed to 5 mg/m^3^ of BDS 4 h/day for 4 days. Table 3 compares the RNA expression levels of the 4 genes at 4 and 21 days, as well as at 21 days + 10 days recovery that were subsequently analyzed by Western blotting for the 5 mg/m^3^ for 4 days exposure group.

**Table 3:** Time-course of up-regulation of selected genes following inhalation of BDS

Gene	4 days BDS	21 days BDS	21 days + 10 days recovery
*Aldh3a1*	2.43	8.63	1.76
*Cyp1a1*	41.93	22.32	2.14
*Cyp1b1*	30.71	33.10	2.83
*Nqo1*	3.60	7.92	2.16

Data quantified by qRT-PCR are expressed as fold increase compared to their respective control groups

#### Pathway analysis

**Fig. 9:**
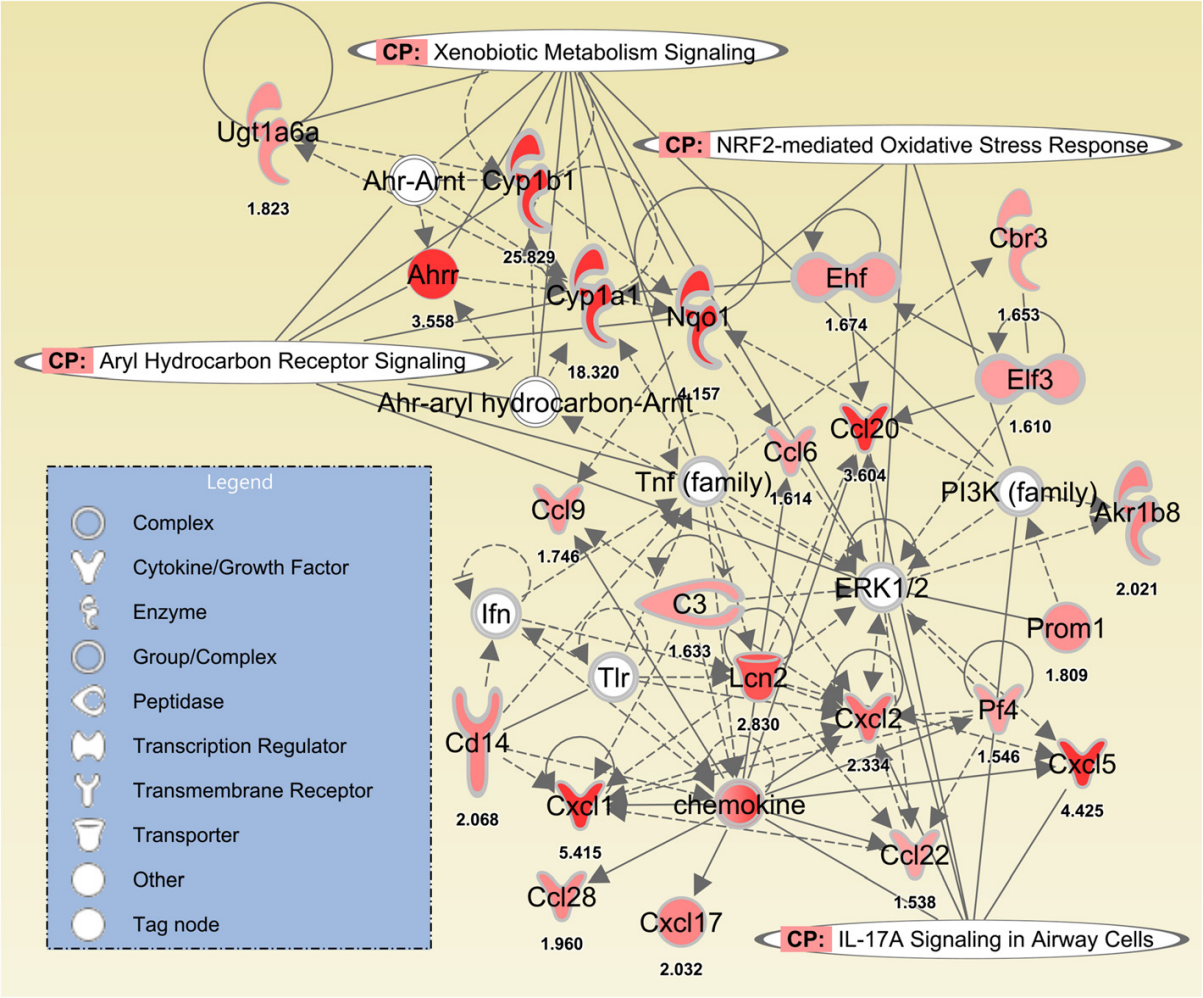
BDS inhalation/exposure (for 3 weeks, with or without 10 days of recovery) predominantly activates/up-regulates genes associated with xenobiotic metabolism, aryl hydrocarbon receptor activity, Nrf2-mediated oxidative stress responses and IL-17A signaling in airway cells, according to Ingenuity Pathway Analysis

Figure 9 presents a network created in the Ingenuity system illustrating the interaction of gene pathways in BDS 21d vs. control mice. The three sets of up-regulated genes identified above - biotransformation, oxidative response and inflammatory/immune - were identified as being differentially expressed and inter-related within specific gene pathways.

## Discussion

Long-term health consequences of involuntary exposure to petrochemical-derived soot particles have received insufficient attention. Populations living in close proximity to heavy road traffic areas, people exposed to refinery, fuel depot and pipeline explosions/fires and even those routinely exposed to refinery flares might be expected to carry a PAH-enriched lung particle burden that would persist after exposures end and activate PAH-metabolism pathways, causing up-regulation of oxidative stress and inflammatory pathways, resulting in histopathological changes to the lungs. In the absence of future exposures, these changes ultimately may be resolved. However, in infants and young children or in individuals with impaired lung function due to chronic diseases (e.g., asthma or COPD) or respiratory infections, symptoms or illness could be aggravated as a result of these additional and largely unavoidable PM exposures. Adverse respiratory effects in healthy and susceptible populations of all ages have been reported at PM exposure levels at or below ambient air quality health standards [42, 43]. The experiments described here were designed to assess a) the histopathologic and transcriptome changes associated with an extended (21 consecutive days) exposure to PAH-rich soots and b) determine how well the changes resolve over time. The 5 mg/m^3^ concentration that was selected for this study is of the same order of magnitude as the levels measured more than one week after the World Trade Center attack (1 mg/m^3^) [14] and five months after the Kuwaiti oil fires of 1991 (830 μg/m^3^) [13].

The BDS aerosols produced in the present study were PM mixtures composed of nanoparticles that aggregated into coarse and fine particles of respirable size (Fig. 1). BDS or petrochemical soots generally cause respiratory effects similar to those caused by other types of air pollutants, such as diesel exhaust particles (DEP) or residual oil fly ash (ROFA), mainly through mechanisms associated with inflammation and oxidative stress [40, 44–47]. Despite similarities with DEP and ROFA for particle sizes and PAH distribution, BDS is relatively low in oxygen and metals content [38]. Since numerous studies [48–52] have associated the PAH content adsorbed onto PMs as a key contributor to PM-induced adverse health effects, including childhood asthma [51] and systemic inflammation [53], the concentration of PAHs, particularly pyrene, acepyrene, anthracene and fluoranthene on the PM in the present study are implicated in the observed activation of PAH-metabolism pathways [38] (Figs. 6, 7, 8 and 9). These results show that conducting both physical and chemical characterization of the aerosol is essential to adequately correlate the lung responses to the airborne PM mixtures used.

Since inhalation is the main portal of entry for PMs, the fate and toxicity of these particles are dictated by the lungs’ deposition and clearance mechanisms [54]. Effective elimination of particles deposited in the lungs is largely accomplished by alveolar macrophages and by mucociliary clearance [55–57]. Limited translocation however, of fine and ultrafine insoluble particles from the lungs to extra-pulmonary organs, such as the heart, has been reported [58–60]. In the present study, although extra-pulmonary translocation may have ensued, the lung responses and recovery following sub-acute exposures to BDS were the focus and extra-pulmonary translocation was not investigated.

Recently, macrophages filled with carbon particles have been investigated as a potential novel biomarker of combustion-derived PM exposures in humans [61]. That systematic review showed that for exposures to combustion-derived PM_10_, ranging from 20 – 30 μg/m^3^, carbon load in macrophages reflected cumulative exposure and was appropriate for personal exposure estimates. A study conducted in children found that the median area of black material (i.e. carbon) in alveolar macrophages could serve as a biomarker of past low PM_10_ exposures ranging between 0.15 and 2.5 μg/m^3^ [62]. Although in our study we used BDS as a surrogate for PM_2.5_, approximately 3.2 % of the aerosols were composed of PM_10_ (Fig. 1b). It is unknown whether the BDS we observed in lung macrophages 10 days post-exposure were filled with large, i.e. 10 μm, or smaller, i.e. 2.5 μm particles; however, since the majority of the aerosols were composed of PM_2.5_, our data are supportive of future research in animals or in humans on macrophage carbon load as a potential sensitive biomarker for short and long-term exposures to combustion-derived PM_2.5_. Further *in vivo* work should quantify the particles, i.e. carbon, metals and PAH content in the collected macrophages and lung tissue to associate a quantitative value to this biomarker. Furthermore, while BDS-filled macrophages were observed in the lungs 10 days post-exposure, our results also show that alveolar phagocytosis and mucociliairy clearance were not completely effective in the removal of particles at this time point (Fig. 3b). The presence of PM in lung parenchymal cells and macrophages at 10 days after exposure ends is not an unusual adverse effect, given the clearance half-time of poorly-soluble particles in the lungs of mice. Indeed, Bermudez et al. [63] showed that the clearance half-time was 40 days for mouse lungs exposed for 13 weeks to 2 mg/m^3^ of ultrafine TiO_2_ particles. Nonetheless, the potential health risk caused by PM exposures has been associated with persistent particle load in macrophages, inducing subsequent long-term damage in the lungs through irritation and inflammation mechanisms [5, 40]. In addition, we observed the presence of soot particles in the airways, adjacent to epithelial cells (Fig. 3), that were not taken up by macrophages even at 10 days post-exposure. This effect shows that the first steps in the alveolar-macrophage mediated clearance, i.e., the detection and recognition of the deposited particles by the alveolar macrophages, is incomplete at 10 days post-exposure. Numerous studies have noted that the phagocytic activity of alveolar macrophages is optimal for particles larger than 500 nm and less effective for particles in the ultrafine (<100 nm) range [34, 36, 58, 60, 64, 65]. It is thought that given their small size, ultrafine particles do not produce signals strong enough to stimulate macrophage chemotaxis towards their target [66–68]. Thus, our data show that even though the overall acute lung responses to BDS inhalation were modest, the potential exists for long-term effects through persistent particle load and free particle interaction with the airways.

IL-1β is an acute phase cytokine associated with macrophage activation and inflammation [69–71]. In addition, activated macrophages can release numerous other inflammatory mediators which attract other immune cells, such as neutrophils, to the site of the injury [69–71]. Figure 2 shows that the percentage of neutrophils in BALF was significantly increased in the BDS 21d group compared to the respective air control and BDS 21d + 10d recovery groups, suggesting that the overall inflammatory reaction process was initiated during exposure, but returned to baseline values after the exposure ended. Up-regulation of the IL-1β gene has previously been reported in both *in vitro* and *in vivo* studies of PM associated with PAHs [40, 50, 52]. We have previously reported that levels of IL-1β were significantly increased in BALF of mice exposed to 5 mg/m^3^ of BDS for 4 days vs. air-control mice [40]. In the present study, since IL-1β was significantly elevated in the BALF of mice exposed to BDS for 21 days (Fig. 5), this result suggests that the sub-acute inhalation exposure to combustion-derived particles caused an inflammatory reaction in the lungs (Figs. 2 & 4). Hence, even though the lung inflammatory response seemed modest after 21 days of exposure to BDS, our data, with increased levels of IL-1β in BALF, indicate local airway damage.

Based on the histopathology score, mucosal inflammation was the most severe lung response to 21 days of exposure to BDS (Fig. 4c). Recovery, however, was observed 10 days post-exposure for mucosal inflammation and epithelial damage, but not for neutrophilic and interstitial inflammation (Fig. 4). This shows that different regions within the lung repair at different rates, with inflammation located in the mucus membranes resolving faster than inflammation located in the connective tissues. Thus, one conclusion from this study is that mice are capable of recovering from mucosal inflammation. Total lung recovery, if it occurs, probably requires a longer period than 10 days in mice. According to an epidemiology study on WTC-exposed firefighters who were present at ground-zero starting immediately after the attack, only 36.4 % had recovered from lower-respiratory symptoms, including cough, dyspnea and wheeze, 7 to 9 years after the attack [72]. This study in humans showed that after acute exposures to high concentrations of PM, resolution of low-respiratory symptoms was possible for a moderate percentage of the cohort. The BDS results presented here suggest that lung mucosal inflammation recovery is one of the first pulmonary injuries to resolve.

It is well known that conducting gene expression analysis on collected tissues provides a specific time window on changes occurring at the RNA and corresponding protein levels [52]. These temporal changes can evolve quickly and thus, assessing gene expression analysis at more than one time point is crucial for obtaining a global picture of the processes [52]. In the present study, gene expression analysis was performed at two time points, immediately after the 21-day exposure ended and following a recovery period of 10 days. For selected proteins, levels were quantified after 4 days of exposure. This allowed evaluation of the time-course and extent of pulmonary recovery following a sub-acute exposure to 5 mg/m^3^ of BDS.

Among the genes up-regulated in the 21-day BDS group (Table 1, Fig. 6) were *Cxcl1* (5.42 fold), *CxCl2* (2.33 fold) and *Cxcl5* (4.43 fold), which are all involved in neutrophil chemotaxis [69–71, 73]. Up-regulation of these 3 genes in the 21-day BDS group supports the BALF differential (Fig. 2) and the histopathology results (Fig. 4A) which highlight the presence of neutrophils in lungs of the 21-day BDS group. This same set of genes was also up-regulated to a similar extent in the lungs of mice exposed to BDS for 4 days [40], but not in lungs of the 21-day BDS + 10 days of recovery group (Table 2; Figs. 2 and 7). These results indicate that the pulmonary inflammatory responses are acute reactions to soot nanoparticles and suggest that the soot components responsible for eliciting the lung inflammatory responses are labile.

In addition to inflammation-related genes, 21 days of exposure to BDS significantly elevated the gene expression levels of *Ahrr* (3.56 fold), *Nqo1* (4.16 fold), *Aldh3a1* (7.97 fold), *Cyp1a1* (18.32 fold) and *Cyp1b1* (25.83 fold), all of which are related to biotransformation and oxidative stress (Table 1, Fig. 6). Ten days after BDS exposures ended, many of the genes that were up-regulated in lungs of BDS 21d mice (above) remained up-regulated although the absolute levels of expression were reduced significantly, up to 90 % (Table 2; Fig. 7). Phase I biotransformation genes in this group included *Ahrr* (2.14 fold) and *Cyp1b1* (2.60 fold); the inflammatory/immune response genes *Il*-*6* (2.08 fold) and *Cxcr6* (2.22 fold); and the acute phase proteins *Orm2* (2.17 fold) and *Saa1* (1.69 fold). In the lungs, PAH metabolism and detoxification pathways usually process through the cytochrome p450 systems, particularly the isoforms *Cyp1a1*, and *Cyp1b1* [52]. Both of these isoforms are of interest since via activation of the aryl hydrocarbon receptor (*Ahr*) they metabolize PAHs from PM, which can lead to bio-activation of metabolites [49, 52, 74–76]. Supporting our results are previous studies conducted in mice exposed to various types of PM that have reported increased gene expression of *Cyp1a1* and *Cyp1b1* in the lungs [40, 52, 77, 78]. In our study, even 10 days after BDS exposures ended, 6 core BDS genes (*Ahrr*, *Cyp1a1*, *Cyp1b1*, *Nqo1*, *Il*-*6*, Ptgs2) related to biotransformation, oxidative stress and inflammation remained up-regulated in the lungs of BDS-exposed mice (Fig. 7). Also, following 21 days of exposure to BDS, the expression of phase I biotransformation genes (*Cyp1a1* and *Cyp1b1*) was much higher (>22 fold increase) than the expression of phase II biotransformation genes (*Aldh3a1* and *Nqo1*) (8 to 9 fold increase; Table 3). However, after a 10-day recovery period, the expression of these phase I and II biotransformation genes returned to similar levels of expression (≈2 fold increase) that were still significantly increased compared to controls (Table 3, Fig. 7). These results indicate that the elevated expression of phase I biotransformation genes is an acute response to soot nanoparticles, and that 10 days after exposure has ended, phases I and II of biotransformation might still be occurring in the lung.

Xenobiotic metabolism and aryl hydrocarbon receptor pathways have been related to PAH biotransformation and elimination processes, both in *in vitro* and *in vivo* studies [40, 41, 52, 74, 75, 77, 78], while oxidative stress and inflammation pathways have mostly been associated with ultrafine particle – cell interactions [79–89]. The latter studies, which used inert ultrafine particles, without any PAH content, such as carbon black and titanium dioxide, showed significant increases in inflammation and oxidative stress *in vitro* and *in vivo*. These studies therefore suggest that the baseline physical effects (inflammation and oxidative stress) induced by inert ultrafine particles in the lungs might be related to the persistent physical presence of the particles [5, 40]. Our gene expression results are supported by the Ingenuity Pathways Analysis, which revealed the relationships connecting the 4 different networks of up-regulated genes, i.e. xenobiotic metabolism, aryl hydrocarbon receptor, Nrf2-mediated oxidative stress responses and IL-17a signaling in airway cells (Fig. 9). These networks link the BDS physicochemical characterization: a) its PAH content [38], to xenobiotic metabolism and Ahr pathways (Table 1, Figs. 6, 7, 8 and 9) and b) the physical presence and size distribution of the BDS aerosols (Fig. 1) [37, 38], to oxidative stress-related responses and inflammation signaling pathways (Table 1, Figs. 2, 3, 4, 5, 6, 7, 8 and 9). Similar networks also were identified in our previous study where mice were exposed to BDS for 4 days [40]. Taken together, these data on responses to inhaled aerosolized BDS indicate that at least two distinct but co-operating processes underlie the lung responses to PAHs adsorbed onto ultrafine particles.

Quantitative qRT-PCR were carried out to confirm the microarray gene expression results from BDS 4 days, BDS 21 days and BDS 21 days + 10 days recovery mice for selected genes, including the attenuated, but still significantly elevated, values in the BDS 21d + 10d recovery group vs. those in BDS 21d group (Figs. 7 and 8). In most cases, the expression fold changes via PCR exceeded the equivalent microarray values. A total of 18 genes were confirmed by PCR. A comparison of the gene expression levels for exposure periods of 4, 21 and 21 days + 10 days recovery is shown in Table 3. *Cyp1a1* gene expression level decreased somewhat, but remained significantly elevated, from 4 to 21 days of exposure (Table 3) This result suggests an adaptive response for this biotransformation gene. Protein expression of biotransformation related genes, ALDH3A1, CYP1A1, CYP1B1 and the oxidative stress related gene NQO1 were increased in mice exposed to 5 mg/m^3^ of BDS for 4 days compared to their respective filtered-air controls (Fig. 8). Correspondingly, the gene expression fold changes measured by qRT-PCR were up-regulated by more than 2.4-fold compared to the controls in both the 4-and 21-day BDS exposure groups (Table 3). In addition, the gene expression fold changes were higher for *Cyp1b1* than for *Cyp1a1* after 21 days of exposure (Table 3). Similar results comparing protein expression of CYP1A1 and CYP1B1 following PM exposures have been reported previously [74, 75]. In a study conducted by Iba *et al*. [75] CYP1A1 and CYP1B1 activities, related to their catalytic and induction properties, were assessed *ex vivo* in human lung samples. Analysis of these enzymes by western blot showed that benzo[*a*]pyrene (B[*a*]P) and diesel exhaust extract induced CYP1A1, but not CYP1B1. In another study, Courter *et al*. [74] reported that in mouse epidermis exposed to urban dust particulate matter, B[*a*]P and dibenzo[*a,l*]pyrene, that CYP1A1 protein expression was increased following individual exposures to all three substances and to co-treatment exposures, while CYP1B1 protein was not expressed in even one of the co-treatment groups. The Ah receptor regulates the expression of these two enzymes, and our results, as well as others from the literature, point out that, at least at the protein level, CYP1A1 seems to be more sensitive to changes induced by PAHs adsorbed onto PM than is CYP1B1. Overall, our microarray, qRT-PCR and western blotting results support the conclusion that 21 days of exposure to BDS induces a transient, time-dependent up-regulation of *Ahr*, biotransformation and oxidative stress related genes. Another study has also shown that changes in gene expression were transient and suggested lung recovery in mice following exposures to approximately 94 μg/m^3^ of urban air pollution [90]. In that study, a 3-week exposure resulted in changes reflected through 70 differentially-expressed genes compared to HEPA-filtered air control mice, while 6 weeks after a 10-week exposure to those same particles had ended, the number of differently expressed genes, which were associated with metabolism pathways, decreased to 13. Thus, that study and ours suggest that the genes that are persistently altered following a recovery period could be the ones responsible for the long-term lung effects induced by combustion-derived particles. In our study, the genes that were still significantly up-regulated after 10 days of recovery included *Ahrr*, *Cyp1a1*, *Cyp1b1*, *Nqo1*, *Il-6* and *Ptgs2* (Fig. 7). Further studies should investigate the specific role(s) of these genes in chronic cardio-respiratory diseases induced by PM exposures.

Previously we reported that PAHs adsorbed onto the surface of BDS are transferred to the plasma membrane of human bronchoepithelial cells (BEAS-2B) and subsequently to the cell interior [38]. We also reported that the PAHs were accumulated / stored within lipid droplets of these cells, and that the PAHs were able to activate xenobiotic metabolism pathways *via* the smooth endoplasmic reticulum, as evidenced by up-regulation of *Cyp1a1*, *Cyp1b1,* and *Aldh3a1* [41]. Furthermore, mice exposed to 5 mg/m^3^ of BDS for 4 days exhibited airway inflammation; neutrophilia, bronchial and epithelial damage; as well as particle accumulation in alveolar macrophages, accompanied by up-regulation of biotransformation, oxidative stress and pro-inflammatory genes [40]. In the current study, we show that 21 days of exposure of mice to 5 mg/m^3^ of BDS induced results similar to those in our 4-day exposure study [40]. In addition, we report that a recovery period of 10 days, following 21 days of exposure, is insufficient to reduce 1) lung connective tissue inflammation, 2) particle alveolar load, and 3) expression of multiple biotransformation, inflammation and oxidative stress-related genes, to baseline levels. Thus, building upon our previous work, both *in vitro* and *in vivo*, on health effects induced by BDS aerosols [37, 38, 40, 41], we hypothesize that these PM health effects are associated with a “double hit” mechanism, whereby the PAHs’ chemical effect is added to the physical BDS nanoparticle effect. First, PAHs, mostly pyrene, acepyrene, anthracene, and fluoranthene, adsorbed onto the BDS particles, are transferred to the lung cells and localize within lipid droplets, initiating the up-regulation of phase I (*Cyp1a1* and *Cyp1b1*) and later phase II (*Aldh3a1* and *Nqo1*) biotransformation genes (Tables 1, 2 and Fig. 9; [37, 38, 41]). The accumulation and storage of these chemicals in lipid droplets prolong the activation of biotransformation genes, as evidenced by significant up-regulation of these genes even 10 days post-exposure (Fig. 7; [41]). Furthermore, the physical presence of free BDS particles in the lung parenchyma, both immediately after exposures end, as well as 10 days post-exposure, added to persistent particle-laden macrophages (Fig. 3; [40]), produces, *via* the normal particle elimination processes, oxidative stress (Nfr2-related responses), as well as mild and transient inflammatory reactions (IL-17a signaling; Figs. 2, 4 and 9; [40]). As mentioned previously, the damage produced by the chemical and physical components of the BDS share common toxicity pathways. Most importantly, we believe it is the storage of PAHs in lipid droplets, which could extend their presence in the organism well beyond the initial removal of particles by alveolar macrophages from the lungs, that facilitates persistent changes in gene expression, and may contribute to long-term lung or systemic effects induced by BDS. Hence, the incomplete lung recovery following sub-acute inhalation exposures to PAH-rich PM_2.5_ may thereby also promote a pro-Ahr activated, pro-oxidant, and pro-inflammatory milieu in the lungs, which may aggravate prior existing conditions or sensitize this environment to the development of cardio-respiratory diseases, including lung cancer and atherosclerosis.

## Conclusion

In summary, we have shown that sub-acute exposures of mice to fine particles rich in PAHs induced: (1) BALF neutrophilic inflammation; (2) lung mucosal inflammation, and (3) increased BALF IL-1β concentration; with all three outcomes returning to baseline levels 10 days post-exposure. In addition, (4) lung connective tissue inflammation persisted 10 days post-exposure; (5) time-dependent up-regulation of Ahr-biotransformation and oxidative stress genes was detected; and (6) persistent particle alveolar load following 10 days of recovery was observed. Thus, our data show that even though the overall sub-acute lung responses were modest, incomplete lung recovery may be a starting point for potential long-term cardio-pulmonary effects. More research is needed to understand the cardio-pulmonary impact of persistent lung gene expression changes after a recovery period following sub-acute or sub-chronic exposure to combustion-derived particles.

## Methods

### Animal protocols

Six-week old female BALB/c mice were obtained from Jackson Laboratories (Bar Harbor, ME). After a one-week acclimation period, animals were housed individually in suspended steel wire cages at the AAALAC-accredited Inhalation Research Facility at Louisiana State University. The mice were handled in accordance with the NIH *Guide for the Care and Use of Laboratory Animals*; and all procedures were approved by the Louisiana State University Institutional Animal Care and Use Committee. Food and water were provided *ad libitum* between exposures, but were removed during the daily exposures to prevent their contamination with particles and/or chemical residues.

### BDS generation and exposures

BDS was generated as described previously [38]. Briefly, BDS generation took place in a 0.25 m^3^ stainless steel and plexiglass chamber that was connected to an adjacent stainless steel and plexiglass 0.25 m^3^ inhalation chamber in which the animals were exposed to BDS. The BDS was drawn from the generation chamber to the exposure chamber by a static pressure differential. HEPA-filtered air was used to maintain steady-state targeted BDS particle concentrations between 5.0–6.5 mg/m^3^ in the exposure chamber. The 10-day exposure yielded a mean mass concentration and a standard deviation of 6.08 ± 3.0 mg/m^3^. This concentration was selected to address the knowledge gap on health effects induced by short-term exposure to elevated levels of PM in humans, that are representative of real life events, including wildfires, pipeline sabotage and accidents at refineries. HEPA-filtered airflow rates in control and exposure chambers were maintained at approximately 140 L/min. Particle concentration in the exposure chamber was monitored in real-time with a DustTrak (Model 8520; TSI Inc., St. Paul, MN); this concentration was confirmed by gravimetric filter comparison. Both the DustTrak probe and the filter holder were positioned in the airflow immediately above the cages housing the mice. For the size distribution, as described in [38], particles were collected and size-fractionated by a three-stage (<2.5 μm; 2.5–10 μm and > 10 μm) RespiCon virtual sampler. Detailed descriptions of the soot aerosols particle size distribution, PAH and metals content of BDS have been presented previously [37, 38].

In the first part of the experiment, six eight-week old female mice inhaled BDS mixed with HEPA-filtered air (4 h/day, 21 consecutive days), while six female controls were exposed only to HEPA-filtered air for the same time. All mice in each group were euthanized by an intraperitoneal injection of 0.2 ml Beuthanasia-D Special (Schering-Plough, Union, NJ) immediately following exposure on the 21^st^ day. In the second part of the experiment, eight female BALB/c mice were exposed to BDS and seven female mice were exposed to HEPA-filtered air for 21 days as described above, and were allowed to recover for 10 days before they were euthanized. Additionally, we exposed four female BALB/c mice by inhalation to either filtered air or BDS (5 mg/m^3^; 4 h/day; for four consecutive days) to confirm our previously published data [40]. This in turn provided for the present study, a comparison time-point to identify gene expression and related protein expression associated with early pulmonary responses. This allowed us to determine the temporal expression of selected genes and proteins associated with mechanisms related to saturation processes and biotransformation phases.

### Lung sample collection and processing

Following euthanasia, the lungs were lavaged twice with 0.5 mL phosphate-buffered saline passed through a 19-gauge cannula anchored in the trachea. Pooled broncho-alveolar lavage fluid (BALF) was immediately placed on ice. Three-hundred cell differential counts were performed on modified Wright’s-stained cytocentrifuge slide preparations of 400 μL aliquots of raw BALF. Levels of the BALF cytokines IFN-γ, IL-1β, IL-6, IL-10, IL-12p70, KC/GRO/CINC, and TNF-α were assayed with the Mouse ProInflammatory 7-Plex Ultra-Sensitive Kit (Meso Scale Diagnostics, Rockville, Maryland). The lower limit of detection for all the cytokines ranged from 0.38–35 pg/mL. This assay was performed as specified by the manufacturer.

### Histopathology

The right lung of each mouse (that was previously lavaged) was perfused with 0.4 mL of freshly prepared 0.02 M periodate-0.1 M lysine-0.25 % paraformaldehyde (PLP) fixative in phosphate buffer (pH 7.4), then excised and stored in PLP for 24–48 h before standard histological processing, sectioning and hematoxylin-eosin staining. Slides containing sections of 3 major lobes of the right lungs were coded randomly. They were then evaluated by an experienced veterinary pathologist with expertise in pulmonary pathology. Initially, all slides were scored at 20 × and 100 × magnifications to determine overall inflammatory cellularity. In this study, all responses were either normal or with a mild increase in cellularity. Then, eight slides, four normal and four mildly-increased, were evaluated in detail to determine the gradient of changes present in specific anatomical subunits of the lungs. If no gradient could be detected, they were not scored (for example, bronchi and pleura in this study). If an intensity gradient was detected, it was scored on an arbitrary 0–3 scale with 0 = normal, = 1 mild change, 2 = moderate change, 3 = severe change (with overall cellularity not exceeding mild, no severe changes were detected in any subdivision). The approximate values for these scores are as follows:

### Epithelial damage

Normal = none. Mild = 2 or fewer contiguous epithelial cells with cytopathology, attenuation, or sloughing at few, scattered sites. Moderate = 3–10 contiguous epithelial cells with cytopathology, attenuation, or sloughing at few, scattered sites, or frequent mild changes. Severe = multiple denuded areas of bronchiolar/bronchial mucosa with attenuation or regeneration of adjacent epithelial cells or frequent moderate changes.

### Mucosal inflammation (bronchioles)

0 = normal. Bronchus-associated lymphoid tissue (BALT) aggregates were in small clusters in the branch-angles and/or the combination of lymphocytes, plasma cells, and eosinophils did not exceed ~10 per linear, 600× field. 1 = mild increase. BALT aggregates were larger or rare outside the branch-angles and/or lymphocytes, plasma cells and eosinophils exceeded ~10 per linear 600× field but did not form clusters. 2 = moderate increase. BALT aggregates outside the branch angles were seen in multiple areas and/or lymphocytes, plasma cells and/or eosinophils were seen in clusters in the submucosa. 3 = severe increase. Large BALT aggregates common along bronchioles and/or diffuse infiltrates of lymphocytes, plasma cells, and eosinophils.

### Neutrophils

0 = normal. Neutrophils did not exceed 1–2 per linear, 600× field. 1 = mild increase. Neutrophils were 2–10 per linear 600× field but did not form clusters. Transmucosal exocytosis was rare. 2 = moderate increase. Neutrophils exceeded 10 per linear 600× field and were frequently seen in clusters in the submucosa. Transmucosal exocytosis evident but not widespread. 3 = severe increase. Diffuse infiltrates of neutrophils were present and transmucosal exocytosis was common. Similar parameters were established for interstitial and alveolar inflammation.

### RNA isolation

The excised left lung from each mouse (that was previously lavaged) was preserved in RNAlater (Applied Biosystems, Foster City, CA). Lungs were then removed from the RNAlater solution, and after gentle blotting, were placed into a separate clean 2 mL microcentrifuge tube with 1 mL TRIzol Reagent (Invitrogen, Carlsbad, CA) and a 4.5 mm copper-coated bead. The lung tissue was homogenized with two 2-min 25-Hz passages on a Mixer Mill MM300 (Qiagen, Valencia, CA). RNA was purified from the aqueous phase of the lung homogenate with the Qiagen RNeasy Mini Kit, including RNase-free DNase treatment, according to the manufacturer’s protocol. We measured RNA concentrations with a NanoDrop ND-1000 Spectrophotometer (Wilmington, DE). Values generated from the NanoDrop for all samples fell into the following ranges: 260/280 ratio: 2.10–2.18; 260/230 ratio: 2.01–2.26; concentration: 1056–2969 ng/μL. RNA quality and integrity was assessed for 1:5 dilutions of each lung sample with the Agilent RNA 6000 Nano Assay Kit and the Agilent 2100 BioAnalyzer (Santa Clara, CA). All samples fell within the following ranges: 28S/18S ratio: 1.0–1.7; RNA integrity # 7.8–9.7. Total RNA was converted to cDNA with a High Capacity cDNA Archive Kit (Applied Biosystems; Foster City, CA) according to the manufacturer’s protocol.

### Microarray assay

MouseGenome 430 2.0 Arrays (Affymetrix, Santa Clara, CA) representing > 39,000 transcripts with > 45,000 probe sets were used to assess global gene expression individually in the left lungs from each of four mice/group. Samples were selected for microarray assay based on RNA concentration and on the scores from the Agilent BioAnalyzer results above. The arrays were processed at the Research Core Facility of Louisiana State University Health Science Center-Shreveport.

Double-stranded cDNA synthesized from total RNA was used to create cRNA, which was biotinylated, fragmented, and added to a hybridization cocktail that included probe array controls, bovine serum albumin, and herring sperm DNA. This cocktail was hybridized (16 h; 45 °C) to oligonucleotide probes on microarrays in a GeneChip Hybridization Oven 640. Immediately after hybridization, the array underwent an automated washing and staining protocol on a GeneChip Fluidics Station and was scanned with a GeneChip Scanner 3000. Collection and processing of initial raw data were performed by a GeneChip Workstation. All gene chips and instrumentation were from Affymetrix (Santa Clara, CA). All data collected and analyzed here adhere to the guidelines for Minimal Information About a Microarray Experiment (MIAME).

### Gene expression analysis

The .*cel* files from the GeneChip Workstation data were processed by Expression Analysis Systems (Durham, NC). A principal component analysis was generated to assess clustering of experimental units. Mal performing probes were removed from the data set by REDI (reduction of invariant probes) and a false discovery rate (FDR) was determined by PADE (permutation analysis for differential expression). Expression data for each transcript including transcript *p*-value, FDR, fold change, Affymetrix probe ID, gene symbol and function were recorded. Gene expression clusters were analyzed with DAVID [91]. Heatmaps were produced using CIMminer of the Genomics and Bioinformatics Group, Developmental Therapeutics Branch (DTB), Developmental Therapeutics Program (DTP), Center for Cancer Research (CCR), U.S. National Cancer Institute (NCI).

### Pathway analyses

Gene expression data were analyzed with Ingenuity Pathway Analysis (Qiagen, Ingenuity Systems, Redwood City, CA). As described previously [40, 41, 92], the most significantly-enriched gene networks and canonical pathways (*p* < 0.05) were examined with the Ingenuity Analysis Knowledge Database. We further created custom gene networks to demonstrate the connections between the genes and significantly associated functional networks and signaling pathways.

### Quantitative real time RT-PCR

Quantitative RT-PCR (qRT-PCR) was performed on cDNA samples from lung homogenates with inventoried TaqMan Gene Expression Assays primer-probe sets (Applied Biosystems) for a selection of genes. cDNA samples from all mice in each group were assayed by qRT-PCR, regardless of whether or not those mouse cDNAs previously underwent microarray analysis. Reaction volumes were 25 μL, and 40 reaction cycles were run for each gene in an Applied Biosystems 7300 Real-Time PCR System. Relative gene expression was determined by the comparative cycle threshold (ΔΔC_T_) method, with each gene normalized to hypoxanthine guanine phosphoribosyltransferase (Hprt1) expression [93], and then compared to the air controls. Results are reported as fold change over control [(2^-ΔΔCT^)].

### Extraction of proteins

Proteins were extracted from frozen lung tissue (stored at -80 °C) collected from experimental and control animals, exposed to either HEPA-filtered air or 5 mg/m^3^ of BDS for 4 h a day for 4 days. Tissues placed inside aluminum foil were snap-frozen in liquid nitrogen, mechanically broken by light hammering, and quickly placed into 2 mL round bottom micro-centrifuge tubes containing 300 μL of RIPA lysis buffer (Santa Cruz Biotechnology, Dallas, TX, USA), and three 2.5 mm zirconia/silica beads (Biospec Products Inc.). Tissues were lysed completely with the aid of TissueLyser II (Qiagen, USA) at 25 MHz for 2 min. The lysed tissue was centrifuged at 13,000 g for 5 min at 4 °C. Proteins in the supernatants were aliquoted and stored at -80 °C. BCA protein assay kits (Thermo Scientific, Waltham, MA, USA) were used to determine protein concentrations in the lung supernatants.

### Western blotting

Fifteen micrograms of protein extracts from each of eight lung tissues (four from controls exposed to HEPA-filtered air and four from exposed mice that inhaled BDS at 5 mg/m^3^ for 4 h a day for 4 days) were incubated in Laemmli buffer (Bio Rad Laboratories Inc., Hercules, CA, USA) with 2-mercaptoethanol, heated for 95 °C for 5 min in a PCR machine, and resolved by SDS-PAGE on Any kD™ Mini-PROTEAN® TGX™ Gel (Bio-Rad Laboratories, Inc., Hercules, CA, USA) at 100 volts until expected separation of proteins was obtained. Proteins resolved on the gels were transferred onto PVDF membranes (Immobilon-P, pore size of 0.45 μm, Millipore Inc., USA) with the Trans-Blot Turbo™ Trransfer System (Bio-Rad Laboratories, Hercules, CA, USA). Antibodies to β-actin (ACTB), which was used as the control, and NAD(P)H quinone oxidoreductase 1 (NQO1) were purchased from Cell Signaling Technology Inc., (Danvers, MA, USA). Antibodies to cytochrome P450 1A1 (CYP1A1), and cytochrome P450 1B1 (CYP1B1) were from Santa Cruz Biotechnology, Inc., (Dallas, TX, USA). The aldehyde dehydrogenase 3A1 (ALDH3A1) antibody was obtained from Thermo-Fisher Scientific Inc., (Waltham, MA, USA). All antibodies were diluted in blocking buffer made with 1× TBS (Bio-Rad Laboratories) supplemented with 1 % bovine serum albumin (Immucor Inc., Norcross, GA), and 0.1 % Tween 20 (Bio-Rad Laboratories). Immunoblots were detected with the aid of the ECL-Prime Western Blotting Detection reagent (GE Healthcare, UK). Western blot images were captured by with ChemiDoc™ Touch imaging system (Bio-Rad Laboratories, Hercules, CA, USA), and the captured images were analyzed with Image Lab 5.2 (Bio-Rad Laboratories, Hercules, CA, USA). Experiments were run in duplicate or triplicate.

### Statistical analysis

We used UNIVARIATE and TTEST procedures of the SAS statistical package (version 9.1.3; SAS Institute, Inc., Cary, NC) to compare BALF cytokine and qRT-PCR data. We used a folded F-test for each data set to determine if the variances across the set were statistically ‘equal’, in which case the variances could be pooled for determining statistical differences. For the occasional cases of unequal variance across a data set, we used Satterthwaite’s approximation of degrees of freedom to determine statistical significance. We analyzed BALF cytology results by ANOVA, followed by the Tukey test. Statistical significance was achieved when *p* < 0.05.

## Abbreviations

[B(a)P]: benzo(a)pyrene
Ahr: aryl-hydrocarbon receptor
BALF: broncho-alveolar lavage fluid
BALT: bronchus-associated lymphoid tissue
BD: 1,3-butadiene
BDS: butadiene soot
DEP: diesel exhaust particles
PAH: polynuclear aromatic hydrocarbon
PM: particulate matter
qRT-PCR: quantitative real-time polymerase chain reaction
ROFA: residual oil fly ash
WTC: World Trade Center
